# Adsorbent Material Based on Carbon Black and Bismuth with Tunable Properties for Gold Recovery

**DOI:** 10.3390/ma16072837

**Published:** 2023-04-02

**Authors:** Cătălin Ianăşi, Paula Svera (m. Ianăşi), Alexandru Popa, Radu Lazău, Adina Negrea, Petru Negrea, Narcis Duteanu, Mihaela Ciopec, Nicoleta-Sorina Nemes

**Affiliations:** 1 Faculty of Industrial Chemistry and Environmental Engineering, Politechnica University Timişoara, Victoriei Square, No. 2, 300006 Timisoara, Romania; 2 National Institute for Research and Development in Electrochemistry and Condensed Matter, 144th Dr. A.P. Podeanu Street, 300569 Timisoara, Romania; 3 Coriolan Drăgulescu Institute of Chemistry, Bv. Mihai Viteazul, No. 24, 300223 Timisoara, Romania; 4 Renewable Energy Research Institute-ICER, Politehnica University of Timisoara, 138 Gavril Musicescu Street, 300501 Timisoara, Romania

**Keywords:** gold recovery, adsorption, bismuth (III) carbonate basic, carbon black

## Abstract

Adsorption recovery of precious metals on a variety of solid substrates has steadily gained increased attention in recent years. Special attention was paid to the studies on the characterization of the adsorptive properties of materials with a high affinity for gold depending on the nature of the pendant groups present in the structure of the material. The aim of the present work was to synthesize and characterize a new material by using the sol-gel synthesis method (designated as BCb/CB). In this case, synthesis involved the following precursors: bismuth carbonate (III), carbon black, and IGEPAL surfactant (octylphenoxypolyethoxyethanol). Immobilization of the heterojunction as bismuth oxide over a flexible support such as carbon black (CB) can prevent their elution in solution and make it versatile for its use in a system. In this work, a new adsorbent material based on bismuth carbonate supported over carbon black (BCb/CB) was developed and used further for gold recovery from aqueous solutions. The required material was characterized physically/chemically by scanning electron microscopy (SEM); energy dispersive X-ray spectrometry (EDX); X-ray diffraction (XRD); thermal analysis (DTG/DTA); atomic force microscopy (AFM). The Brunauer–Emmett–Teller (BET) method was used to determine the specific surface area indicating a value of approximately 40 m^2^/g, higher than the surface of CB precursor (36 m^2^/g). The adsorptive properties and the adsorption mechanism of the materials were highlighted in order to recover Au(III). For this, static adsorption studies were carried out. The parameters that influence the adsorption process were studied, namely: the pH, the contact time, the temperature, and the initial concentration of the gold ions in the used solution. In order to establish the mechanism of the adsorption process, kinetic, thermodynamic, and equilibrium studies were carried out. Experimental data proved that the gold recovery can be conducted with maximum performance at pH 3, at room temperature. Thermodynamic studies proved that the gold adsorption on BCb/CB material is a spontaneous and endothermal process. The results indicate a total adsorption capacity of 13.1 mg Au(III)/g material. By using this material in real solutions, a recovery efficiency of 90.5% was obtained, concomitant with a higher selectivity (around 95%).

## 1. Introduction

Historically speaking, gold had a huge importance as a currency. In the actual development stage of human society, gold is used as an investment or as raw material for different industries. Gold is part of the precious metal group having a high economic value. Chemically, it tends to have a limited reactivity compared with other chemical elements, but it is usually ductile and glossy [1]. 

Regarding gold production, it was observed that at the end of the 20th century, four countries, South Africa, Russia, the USA, and Australia, produced two-thirds of all world’s gold. At the beginning of the 21st century, China became the world leader in gold production. However, during this period Australia, Russia, the USA, Canada, and South Africa continued to supply large quantities of this precious metal [2,3].

Currently, silver and copper gold alloys are used as raw materials for coins and cutlery production, and alloys with copper, platinum, and palladium are used to make jewelry [4]. Gold thin films are able to reflect up to 98% of incident infrared radiation, being currently involved in satellite construction and the production of space visors, giving them an increased protection degree. Similarly, thin films of gold are used on the windows of large office buildings, leading to a reduction of energy consumption for air conditioning, and it also adds a nice touch [5]. Development of space technology involves gold usage, and right now gold is used in hundreds of ways in every spacecraft that NASA launches due to its specific properties. For example, gold is used in electrical circuit production because it is an efficient and safe conductor and connector. Similar to the plating satellite process, spacecraft are plated with a composite film made of polyester and gold, and they able to reflect infrared radiation, helping with temperature stabilization. Without this specific coating, dark colored parts of the spacecraft would adsorb large quantities of heat, leading to a significant increase of the internal temperature, resulting in negative effects on systems and astronauts. Gold is used as lubricants for the mechanical parts of space shuttles because in a vacuum, classical organic lubricants would volatilize and be broken down by the intense radiation beyond Earth’s atmosphere. This unusual usage is due to gold’s very low shear strength, allowing the gold atoms to pass each other under with little frictional force, providing a lubricating action [6].

Gold has been used in the construction of standard computers because the fast and accurate transmission of digital information through the computer and from one electronic component to another requires an efficient and reliable conductor. Gold fulfills all these requirements better than any other metal. In the manufacture of electrical and electronic equipment, side connectors are used to mount the microprocessor and memory chips on the motherboard, and the plug-and-socket connectors used to attach cables contain gold. Gold from such components is generally electroplated onto other metals and alloys containing small amounts of nickel and cobalt used to increase system durability [7,8,9].

Different methods used for gold recovery can be used only if the total cost is lower than the value of the recovered metal. In addition, restrictions on waste disposal and environmental regulations require the usage of green and economic technologies [10]. In this context, some of the used methods for gold recovery are mechanical recovery [11], pyro metallurgical [12,13,14], hydrometallurgical [15], bio metallurgical [16], percolation [17,18,19,20], cementation [21] processes, solvent extraction [10], ionic exchange [22], adsorption [21], and electrochemical extraction [23].

Adsorption is a viable separation process in which one component or some flux of components from a gaseous mixture, or from a solution are retained onto the surface of the solid adsorbent. This technique has a higher efficiency, has a large availability, and can be easily operated [24]. One of the most important properties of the adsorbent material is represented by its porous structure, being proved that a higher adsorption capacity is related to a higher contact surface due to higher material porosity [25].

In time were developed different adsorbent materials were able to recover gold with higher efficiency, due to the active groups grafted onto the adsorbent material surface. From this category some examples would be vegetable waste, activated carbon, lignin-based gels, hydrogels with different polar groups ((N-(hydroxymethyl) methacrylamide)-1-allyl-2-thiourea), Mn_2_O_3_, chitosan derivatives, cetylpyridine bromide/tributulphosphate, dibutyl carbitol, Cyphos IL-101, 2-hydroxy-4-sec-octanyl diphenyl-ketoxime, tri-n-octylamine/tri-n-butyl phosphate/n-heptane (TBP), methyl isobutyl ketone, tetradecyl-dimethyl chloride-benzyl ammonium, poly (oxyethylene)-9-nonylphenyl ether, Cyanex 923, strong basic macrocyclic triamines, 1, 5, 9-triazacyclododec ani, toluene-tert-octyl-ammonium bromide, tertiary amine chloride, tertiary amine Hostarex A327, 5-dodecyl-licylaldoxime and 2-hydroxy-5-nonyl acetopheonexime + kerosene, non-ionic surfactants, hexanol, methyl-so-butyl ketone, phosphine oxide Cyanex 921, thiourea, and copolymer coP-16 TEDMA/EGDMA (2,2′-thiobisethanol dimethacrylate/ethylene glycol dimethacrylate copolymer) [1]. 

The aim of this study was to prepare a new adsorbent material with managed properties able to be used further for gold recovery by adsorption from aqueous solutions. In this context, bismuth (III) carbonate was inserted into a carbon matrix (using carbon black as a precursor), over which an IGEPAL (octyl-phenoxy-poly etoxy ethanol) surfactant was added to improve the specific surface area of the newly produced adsorbent material. Until now, activated carbon black has been used as adsorbent material for gold recovery due to its important advantages: lower price, higher thermal stability, higher specific surface, a lower sensibility at humidity, and properties that lead to higher adsorption capacities. Carbon black surface modification by loading it with different metallic oxides aims to improve the adsorption capacities of produced materials [26,27]. Until now, synthesized and adsorbent materials used matrix carbon black, which was loaded with calcium oxide [28], lanthanum oxide, silver oxide, titanium oxide, copper oxides, nickel oxide, iron oxides, strontium oxides, or cerium dioxides [28,29,30,31,32,33,34,35,36].

Is known the fact that bismuth (III) oxide has remarkable photocatalytic activity, being used for dye removal from different wastewaters [37,38,39]. Although the activity of the adsorbent materials obtained by carbon black modification with Bi_2_O_3_ used for gold recovery has not been reported in the literature until now, the present study aims to confirm that the newly prepared adsorbent material can be used for gold recovery by adsorption from aqueous solutions.

## 2. Materials and Methods

### 2.1. Materials Synthesis and Characterization

Synthesis of carbon black modified with Bi_2_O_3_ was possible by using a two-stage route. In the first stage, a solution of acetonitrile was prepared by dissolving 10 mL of pure acetonitrile (Merck, Rahway, NJ, USA) in 90 mL of DI water. Further, 10 mL of the obtained solution was mixed with 0.5 g of granulated carbon black (Sigma Aldrich), and mixed for 3 h. After that, the obtained sample was ultrasonicated for 15 min using a QSonica (Q700, Newtown, CT, USA) ultrasonic bath. In the second stage, a solution of bismuth (III) carbonate was prepared by dissolving 0.5 g of carbonate in 5 mL of HNO_3_. The obtained solution was mixed for 2 h. Into the prepared solution was added 1 g of surfactant (IGEPAL), and the obtained solution was mixed for one more hour. Finally, the second solution was mixed with the obtained first solution. The obtained product, designated as RAYPA, was dried at 100 °C for 24 h, and further calcined at 300° for 3 h. 

After preparation obtained materials were characterized by:

− scanning electron microscopy (SEM) using a Quanta FEG 250 microscope (FEI, Hilsboro, OR, USA), in order to get information regarding material surface morphology;
− X-ray energy dispersive spectroscopy—in order to get material elemental composition;
− material specific surface was determined by using Brunauer-Emmet–Teller (BET) method, with a Quantachrome Nova 1200 E system. All samples were degassed under vacuum, at room temperature for 24 h. Adsorption–desorption isotherms were recorded a 77 K under nitrogen atmosphere;
− X-ray diffraction (XRD)—measurements were done using Ultima IV (RIGAKU, Tokyo, Japan) instrument operating with Cu K radiation;
− thermal analysis was performed using a thermos-analyzer system Mettler TGA/SDTA 851/LF/1100. The sample with a mass of about 10 mg was placed in alumina crucibles of 150 μL. The experiments were performed under an air atmosphere with a heating rate of 10 °C/min;
− Atomic force microscopy (AFM), was performed using scanning probe microscopy platform (MultiView-2000 system, Nanonics Imaging Ltd., Jerusalem, Israel).

### 2.2. Gold Recovery by Adsorption Processes

#### 2.2.1. pH Effect

Adsorption studies were conducted by using a pH range between 1 and 14. In this context, pH values were adjusted by using NaOH (0.05 to 2 N) and HNO3 (0.5–2 N) solutions. The gold-containing solution, with a concentration of 10 mg L^−1^, was obtained by dilution of a stock solution having an initial concentration of 1 g L^−1^. Each adsorption experiment was conducted by accurately weighing 0.1 g of adsorbent material (BCb/CB), which further was mixed with 25 mL of the gold-containing solution having a stabilized pH. The obtained samples were shocked using a thermostatic bath for 1 h at 298 K. After that, all samples were filtered, and gold residual concentration was determined by atomic adsorption spectroscopy using a Varian SpectrAAS 280 FS system.

#### 2.2.2. Contact Time and Temperature Effect

In order to determine the influence of contact time and temperature over the maximum adsorption capacity of newly prepared material, exactly 0.1 g of material was mixed with 25 mL of gold-containing solution. The obtained samples were kept in contact with the gold solution for 30, 45, 60, 90, and 120 min. All experiments were carried out in triplicate at three different temperatures (298, 308, and 318 K). After that, all samples were filtered, and residual solutions determined the gold concentration.

#### 2.2.3. Initial Concentration Effect

The effect of initial gold concentration on the maximum adsorption capacity was established by preparing a solution with different gold concentrations (5, 10, 20, 40, 60, 80, 100, 120, and 140 mg L^−1^), followed by mixing 25 mL of gold solutions with 0.1 g of adsorbent material. All obtained samples were kept in contact for 1 h at 298 K at a pH value of 3. The final gold concentration was determined in the residual solution obtained after sample filtration.

### 2.3. Kinetic, Thermodynamic, and Equilibrium Parameters for Adsorption Process

The gold adsorption mechanism was determined by using kinetic, thermodynamic, and equilibrium models. Adsorption isotherm models provides information regarding the possible interactions between the material surface and gold ions from aqueous solutions and data regarding the maximum amount of gold ions adsorbed onto the material surface, being able to establish the interaction mechanism between gold ions and newly prepared adsorbent [40]. Adsorption can take place on a mono or on a multi-layer. 

Adsorption capacity represents the quantity of adsorbed ions retained by the adsorbent material reported at the adsorbent mass or volume unit. The following equation describes the adsorption capacity at equilibrium:

$$q_{e}=\frac{\left(C_{0}-C_{e}\right)V}{m}$$

where: $q_{e}$ is the equilibrium adsorption capacity (mg g^−1^);

$C_{0}$ is the Au(III) initial concentration (mg L^−1^);

$C_{e}$ is the Au(III) equilibrium concentration (mg L^−1^); 

V is the Au(III) solution volume (L);

M is the adsorbent mass (g)

Kinetic studies are used to elucidate the adsorption process mechanism, chemical reactions which are taking place, speed reaction, mass transfer coefficient, and optimum conditions for the studied adsorption process. Most used kinetic models are pseudo-first-order and pseudo-second-order models [41].

Thermodynamic parameters involved in the adsorptive process are free Gibbs energy, standard enthalpy and entropy, being related to energy changement during the adsorptive process [42].

#### 2.3.1. Kinetic Study

Kinetic studies are performed in order to obtain information regarding optimum adsorption condition, adsorption mechanism, and adsorption speed (being able to evaluate mass transfer processes and the chemical reactions which are taking place).

In order to better understand the gold adsorption process and the kinetic mechanism involved, obtained experimental data were modeled using Lagergren (pseudo-first-order) kinetic model and Ho-McKay (pseudo-second-order) kinetic model. Intraparticle diffusion was established by modeling experiment data with Weber and Morris model. All studies were carried out at three different temperatures (298, 308, and 318 K).

From line equation $\ln\left(q_{e}-q_{t}\right)=f(t)$ and $t/q_{t}=f(t)$ were calculated $k_{1}$ and $k_{2}$ speed constants, and $q_{e,calc}$ adsorption capacities for pseudo-first-order and pseudo-second-order kinetic models. The obtained kinetic parameters established which model better describes the studied adsorption process, taking in account the value of correlation coefficient, $R^{2}$.

The kinetic models used are described by following equations:

Pseudo-first-order kinetic equation (Lagergren model) [43]

$$\ln\left(q_{e}-q_{t}\right)=\ln q_{e}-k_{1}t$$

Pseudo-second-order kinetic equation (Ho and McKay model) [44,45]

$$\frac{t}{q_{t}}=\frac{1}{k_{2}q_{e}^{2}}+\frac{1}{q_{e}}$$

where: $q_{e}$, equilibrium adsorption capacity (mg g^−1^);

$q_{t}$ is the adsorption capacity at t time (mg g^−1^);

$k_{1}$ is the pseudo-first-order speed constant (min^−1^);

$k_{2}$ is the pseudo-second-order speed constant (g mg^−1^∙min^−1^);

T is the, contact time (min). 

In order to evaluate if the intraparticular diffusion represents a speed determined stage, the obtained experimental data were modeled using the Weber and Morris model [46,47]:

$$q_{t}=k_{dif}\times t^{0.5}+C$$

where: $q_{t}$ is the adsorption capacity at *t* time; 

$k_{dif}$ is the intraparticle diffusion speed constant (mg/g∙min^−0.5^);

C is a constant correlated with the thickness of the surrounding liquid film adsorbent particles. 

Also, it is possible to establish if the adsorption process is taking place in several stages from graphical dependence $q_{t}=f(t^{1/2})$. Based on that, parameters $k_{dif}$ and C were calculated, establishing whether or not the obtained lines pass through the origin.

The Elovich model is used to describe adsorptive processes which follow the pseudo-second-order kinetics. This model starts from the assumption that the entire adsorbent surface is energetically heterogenous, being applied for chemisorption elucidation [48,49]. The mathematic expression of the Elovich model is:

$$q_{t}=\frac{ln(\alpha \beta )}{\beta }+\frac{{ln}_{t}}{\beta }$$

where:

$q_{t}$ is the adsorption capacity at time *t* (mg g^−1^);

α is the initial adsorbate adsorption rate (mg g^−1^ min^−1^);

β is the adsorption constant (mg g^−1^ min^−1^) [50].

#### 2.3.2. Thermodynamic Studies

Thermodynamic studies were carried out in order to establish energetic changes, which are taking place during gold adsorption, in a temperature range between 298 and 318 K. Based on thermodynamic studies, we can conclude if the studied processes are spontaneous or not and if they are endothermal or exothermal. Such evaluations can be done from the values of free Gibbs energy $\Delta G^{0}$, free entropy $\Delta S^{0}$, and free enthalpy $\Delta H^{0}$. Dependence between these parameters are expressed by the van’t Hoff equation [51]:

$$\Delta G^{0}=\Delta H^{0}-T\Delta S^{0}$$

where: $\Delta G^{0}$ is the standard Gibbs free energy change (kJ mol^−1^);

$\Delta S^{0}$ is the standard adsorption entropy change (J mol^−1^∙K^−1^);

$\Delta H^{0}$ is the standard adsorption enthalpy change (kJ mol^−1^);

T is the absolute temperature (K).

Free entropy and free enthalpy values are evaluated from the linear dependence between $lnK_{d}=f(1/T)$, where:

$$lnK_{d}=\frac{\Delta S^{0}}{R}-\frac{\Delta H^{0}}{RT}$$

where: $K_{d}$ is the equilibrium constant;

$\Delta S^{0}$ is the standard adsorption entropy change (J mol^−1^∙K^−1^);

$\Delta H^{0}$ is the standard adsorption enthalpy change (kJ mol^−1^);

T is the absolute temperature (K);

R is the ideal gas constant (8314 J mol^−1^∙K^−1^).

The adsorption process equilibrium constant represents the ratio between adsorption capacity obtained at equilibrium ($q_{e}$) and equilibrium concentration ($C_{e}$):

$$K_{d}=\frac{q_{e}}{C_{e}}$$

Further free Gibbs has been calculated by using the van’t Hoff equation. 

#### 2.3.3. Activation Energy

The activation energy ($E_{a}$) offers information regarding how the adsorption process is taking place, which could be chemical or physical adsorption [52]. The activation energy was calculated from the Arrhenius equation, by using the speed constant ($k_{2}$) obtained from the pseudo-order-two kinetic model, by using the following equation:

$$\ln k_{2}=\ln A-\frac{E_{a}}{RT}$$

where: $k_{2}$ is the speed constant (g min^−1^∙mg^−1^);

A is the Arrhenius constant (g∙min mg^−1^);

$E_{a}$ is the activation energy (kJ mol^−1^);

T is the absolute temperature (K);

R is the ideal gas constant (8.314 J mol^−1^∙K^−1^).

Activation energy can be seen as the minimum kinetic energy which the reactants must have to promote the chemical transformations needed for adsorption at the liquid–solid interface. The adsorption mechanism can be understood by evaluating the intermolecular forces which determine the process evolution [53].

#### 2.3.4. Equilibrium Study: Isotherms Models

The adsorption mechanism can be identified by describing how the aqueous solution containing gold ions interact with adsorbent material [54]. Such interactions can be described by using equilibrium isotherms, which shows the dependence between quantity of substance adsorbed on one gram of adsorbent material at equilibrium ($q_{e}$) and residual gold ions concentration ($C_{e}$).

Description of studied adsorption was possible by modeling obtained experimental data by using three different adsorption isotherms: Langmuir, Freundlich, and Sips isotherms. 

Non-linear form of Langmuir isotherm is [55]:

$$q_{e}=\frac{q_{L}K_{L}C_{e}}{1+K_{L}C_{e}}$$

where: $q_{e}$ is the equilibrium adsorption capacity (mg g^−1^);

$q_{L}$ is the Langmuir maximum adsorption capacity (mg g^−1^);

$K_{L}$ is the Langmuir constant;

$C_{e}$ is the Au(III) equilibrium concentration (mg L^−1^);

$R_{L}$ represents a non-dimensional constant, which represents the basic feature of Langmuir isotherm, also called the separation factor, or equilibrium parameter. The separation factor can be calculated using the following equation:

$$R_{L}=\frac{1}{1+K_{L}C_{o}}$$

where: $R_{L}$ is the separation factor; 

$K_{L}$ is the Langmuir constant (L mg^−1^); 

$C_{o}$ is the Au(III) initial concentration (mg L^−1^).

The Freundlich isotherm is used to describe the adsorptive process which is taking place on the heterogeneous surface. The Freundlich isotherm empirical equation defines the heterogeneous surface of adsorbent material, the exponential distribution of active centers, and the value of their energy [56]. The non-linear form of the Langmuir isotherm is:

qe=KFCe1nF

where: $q_{e}$ is the equilibrium adsorption capacity (mg g^−1^);

$C_{e}$ is the Au(III) equilibrium concentration (mg L^−1^);

$K_{F}$ and $n_{F}$ are the characteristic constants that can be associated with the relative adsorption capacity of the adsorbent and the adsorption intensity, respectively. The n value indicates the non-linearity degree between solution concentration and the adsorptive process: *n* = 1 means that the studied adsorption is a linear one; when *n* < 1 means that the studied adsorption is a chemical process, and when *n* > 1,the studied adsorptive process is a physical one. It was demonstrated that when *n* is between 1 and 10, we get good physical adsorption [57].

The Sips isotherm [58] represents a combination between the Langmuir and Freundlich isotherms, expressed by non-linear equation:

$$q_{e}=\frac{q_{S}K_{S}C_{e}^{1/n_{S}}}{1+K_{S}C_{e}^{1/n_{S}}}$$

where: $q_{S}$ is the maximum adsorption capacity (mg g^−1^);

$K_{S}$ is the constant related to the adsorption capacity of the material with adsorbent properties;

$n_{S}$ is the the heterogeneity factor.

For lower concentrations of adsorbent, the adsorption process was modeled by Freundlich isotherm, and for higher concentrations, the adsorption process was modeled by Langmuir isotherm [59].

Based on the Sips isotherm, parameters can be calculated a separation factor, which represents a dimensionless equilibrium parameter, by using the following equation:

(1) $$R_{S}=\frac{1}{1+K_{S}C_{0}^{1/n_{S}}}$$

where: $R_{S}$ is the separation factor;

$K_{S}$ is the constant related to the adsorption capacity of the adsorbent;

$n_{S}$ is the the heterogeneity factor;

$C_{0}$ is the Au(III) initial concentration (mg L^−1^).

The separation factor value indicates the adsorption type, being an essential characteristic for the Sips isotherm. R_s_ > 1 indicates that the studied adsorption is not a favorable process with isotherms having a concave shape; when R_s_ = 1, Sips isotherm have a linear shape; and if R_s_ < 1, Sips isotherm have a convex shape, indicating that the studied adsorption process is a favorable one. A separation factor of 0 indicates that the studied adsorption process is an irreversible one.

Sips isotherms are obtained from a graphical representation of dependence $q_{e}=f(C_{e})$, and specific parameters for each isotherm used for modeling of experimental data being obtained from the slopes and from the ordonate at the origin.

## 3. Results and Discussion

### 3.1. Material Characterization

#### 3.1.1. Thermogravimetric Analysis, TG

**Figure 1:**
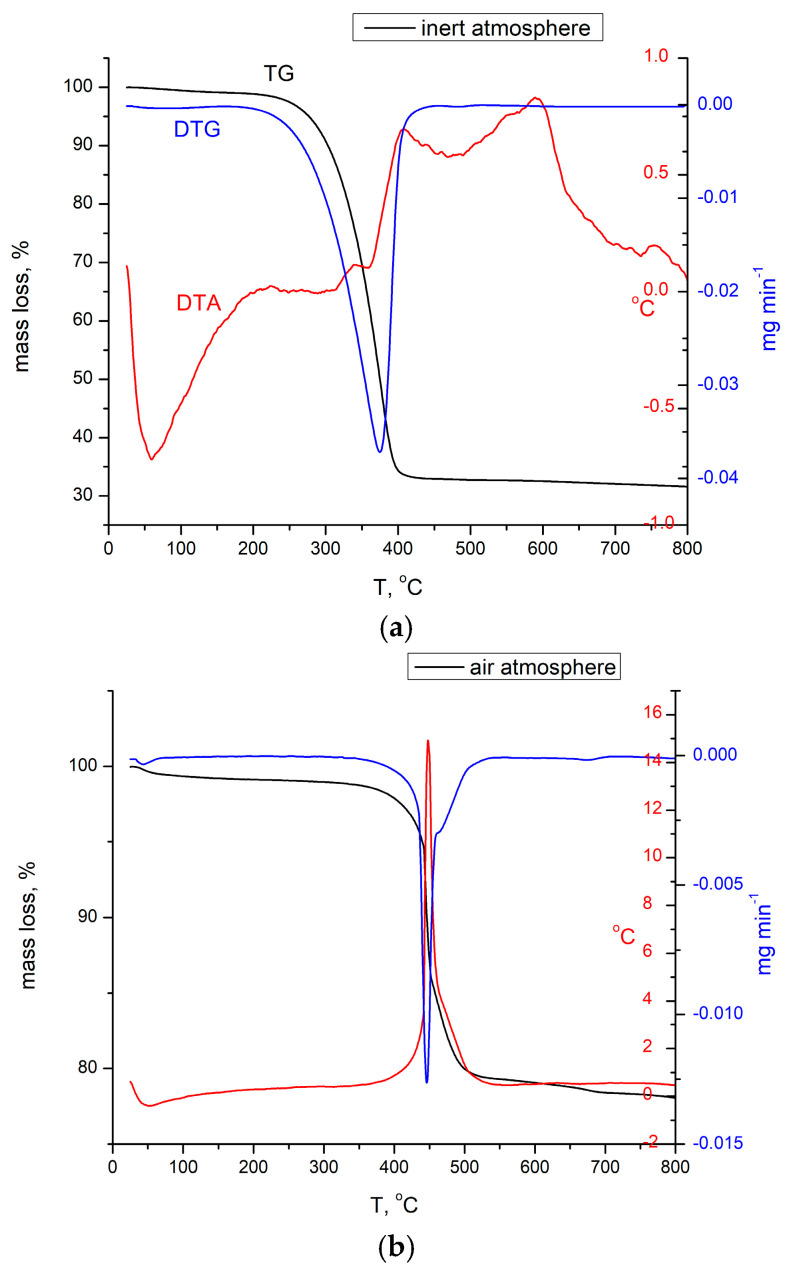
(**a**) TG obtained for sample BCb/CB in nitrogen. (**b**) TG for sample BCb/CB in air.

Figure 1a shows the thermal stability, phase transformation, and composition change of BCb/CB sample from 25 to 800 °C in N_2_ atmosphere with a mass loss of 65%. There was a light loss in the mass of the sample at about 0.5% in the temperature range of 25–200 °C due to dehydration (e.g., moisture release). From 230 to 450 °C, the mass of the sample (with the main compound Bi_2_O_2_CO_3_) changed significantly with a mass loss of 65%, corresponding to the transformation reaction: Bi_2_O_2_CO_3_ → Bi_2_O_3_ + CO_2_. 

The thermal decomposition of (Bi_2_O_2_)CO_3_ in an air atmosphere is a little bit different as the decomposition of basic bismuth carbonate and Bi_2_O_3_ crystallization processes are delayed at 70 °C. 

The decomposition stage of (Bi_2_O_2_)CO_3_ in an air atmosphere was a prolonged weight loss in the temperature range of 300–550 °C, totaling 20.6% of the sample weight (Figure 1b). The decomposition of (Bi_2_O_2_)CO_3_, and the Bi_2_O_3_ crystallization processes are evidenced on the DTA curve by the exothermal peak at 462 °C. As was found out from XRD, the final product of decomposition was alfa Bi_2_O_3_.

#### 3.1.2. X-ray Diffraction (XRD)

**Figure 2:**
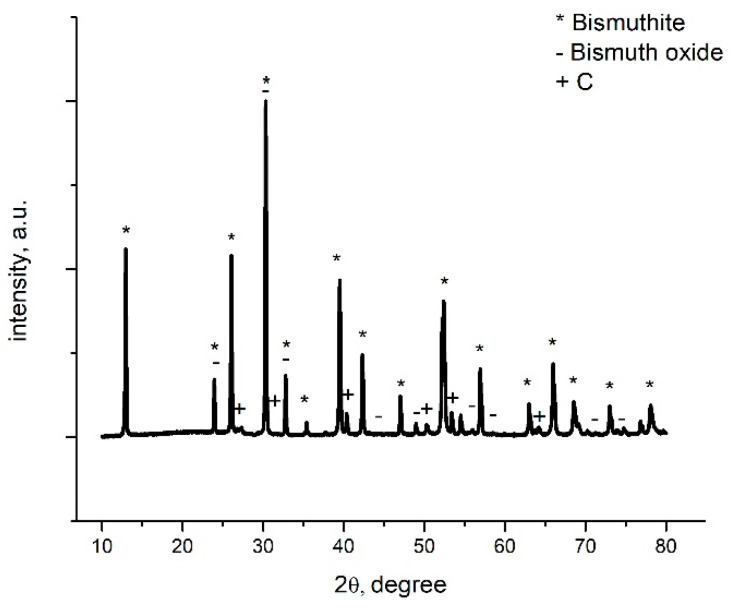
X-ray diffraction spectrum recorded for sample BCb/CB.

Figure 2 presents the XRD spectrum obtained for the newly prepared adsorbent material BCb/BC, which was synthesized at 300 °C for 3 h. From the recorded XRD spectrum, it can be observed that the main phase is represented by bismuthite one, accordingly with JCPS: 00-041-1488, also being observed with one phase specific for bismuth oxide (accordingly with JCPDS: 01-075-0993). Also, it can be observed that the presence of a residual phase is represented by the C introduced in the system during synthesis (accordingly with JCPDS: 00-046-0943). 

The bismuthite presents a tetragonal crystal system with a Space group I4/mmm. Indexed peaks appearing in the recorded spectrum are located at 13, 24, 26, 30, 33, 35, 39, 42, 47, 52, 57, and 63 degrees being assigned to 002, 001, 004, 013, 110, 112, 006, 114, 020, 116, 123, 206 crystalline planes specific to the pure crystal structure of the bismuthites.

Indexed peaks appearing in the recorded spectrum are located at 24, 32, 33, 44, 47, 56, and 58 degrees being assigned to 101, 103, 110, 114, 200, 116, and 213 crystalline planes specific to bismuth oxide.

#### 3.1.3. Scanning Electron Microscopy (SEM) 

**Figure 3:**
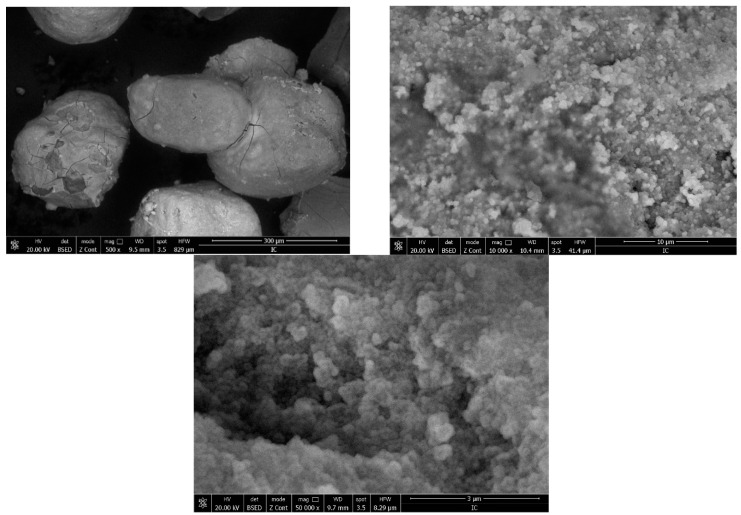
Scanning electron microscope images of sample BCb/CB (500× **left**; 10,000× **right**, 50,000× **second line**).

Figure 3 depicts the SEM images recorded for the newly prepared adsorbent material.

Based on the images presented in Figure 3, one can observe that the prepared adsorbent material has a crystalline morphology. The image recorded at a magnification of 500× shows the presence of some clusters with dimensions of the particles around 250 μm. The image recorded at 10,000× shows that the clusters are formed from overlapped spherical particles, with dimensions around 200 nm. The dimensions are confirmed further by atomic force microscopy (AFM).

#### 3.1.4. Energy Dispersive X-ray Spectroscopy, EDX

**Figure 4:**
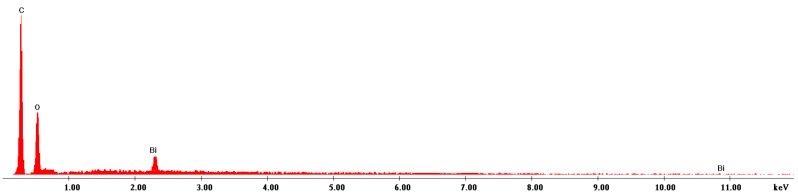
Energy dispersive X-ray spectroscopy, EDX obtained for sample BCb/CB.

Figure 4 presents the EDX spectra recorded for sample BCp/BC used for reliable chemical characterization.

The recorded EDX spectra shows the presence of the specific peaks for bismuth, carbon, and oxygen, confirming in this way the formation of bismuth oxide. Presence of the carbon in the spectra is related to the presence of the bismuth carbonate used during preparation, and to the presence of the carbon band used during the analysis.

#### 3.1.5. Brunauer–Emmet–Teller (BET) Specific Surface Determination

**Figure 5:**
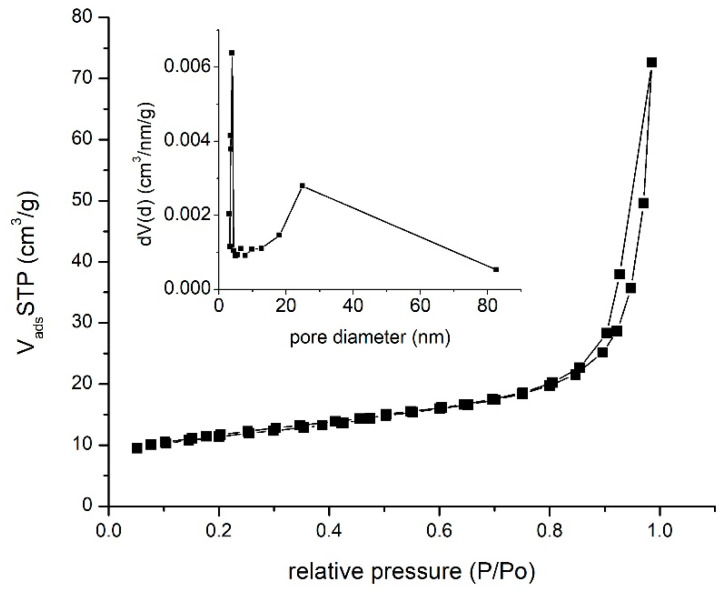
Nitrogen adsorption desorption isotherms obtained for sample BCb/CB. Inset presents the pore size distribution.

Adsorption–desorption isotherms were obtained by recording the N_2_ adsorption–desorption presented in Figure 5. Further, from the data presented in Figure 5, specific parameters were determined by using Barret–Joyner–Halenda (BJH) model. This model is based on the Kelvin equation, establishing a relation between pore radius and the change in the adsorbed/desorbed gas volume at a specific gas pressure.

From the isotherms depicted in Figure 5, we can observe that the studied material presents a type IV with a H3 hysteresis. Information obtained from BET studies confirms the information obtained from SEM analysis. From the recorded isotherm were obtained the parameters presented in Table 1.

In the case of the CB matrix, after carrying out the BET studies, a specific surface of 36 m^2^/g was obtained. Based on these values, it is expected to obtain a higher adsorption capacity in the case of newly prepared adsorbent material.

#### 3.1.6. Atomic Force Microscopy, AFM

The AFM analysis was conducted under ambient conditions (24–25 °C) using the intermittent mode. Before the analysis, the materials were placed on a polished glass slide. A scanner was equipped with a silicone-type probe with a Cr coating and a tip radius of 20 nm with a resonance frequency of 30–40 kHz. 

Calculated values from AFM images (average roughness (Sa), mean square root roughness (Sq), maximum peak height (Sp), maximum valley depth (Sv), and maximum peak-to-valley height (Sy)) are presented in Table 2.

**Figure 6:**
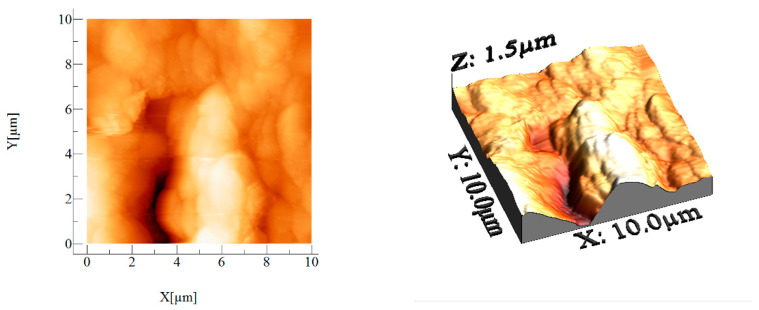
2D and 3D images obtained for BCp/BC sample.

**Figure 7:**
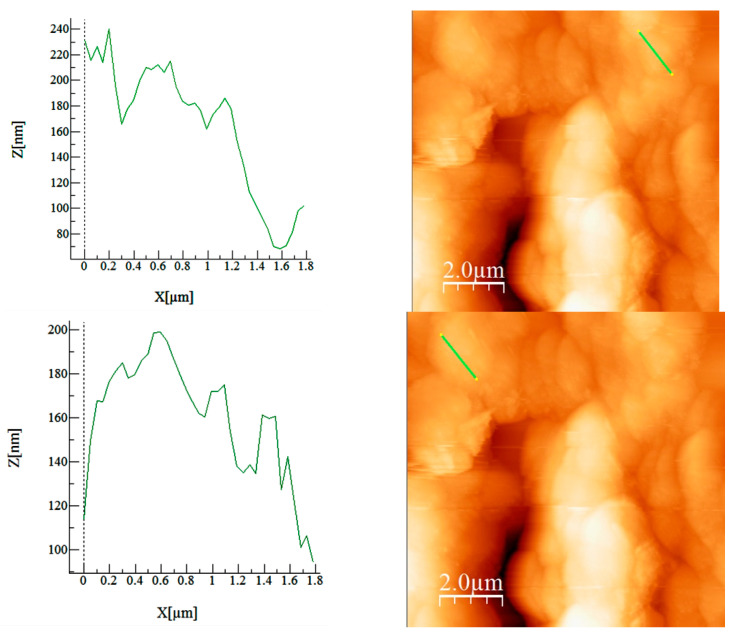
Height and width measurements on the selected area.

Regarding the appearance of the material, the sample shares the presence of rounded agglomerations (Figure 6). Individual areas were measured indicating the height and width of the measured particles (Figure 7). 

The material presents agglomerated nature with different height. The BCp/BC sample indicates high rugosity of the sample, as the more wrinkled is, the more rugous its nature would be and more asperities are registered. Additional confirmation regarding the rugosity of the samples are the Sp, Sv, and Sy values, whereas the Sy value is the sum of Sp (highest peak) and Sv (lowest pit). Greater Sy, greater the rugosity, because the Sy measures the peak-to-peak height, indicating the compactivity of the deposited material or information regarding the surface homogeneity. 

### 3.2. Gold Recovery by Adsorption Processes

#### 3.2.1. pH Effect

**Figure 8:**
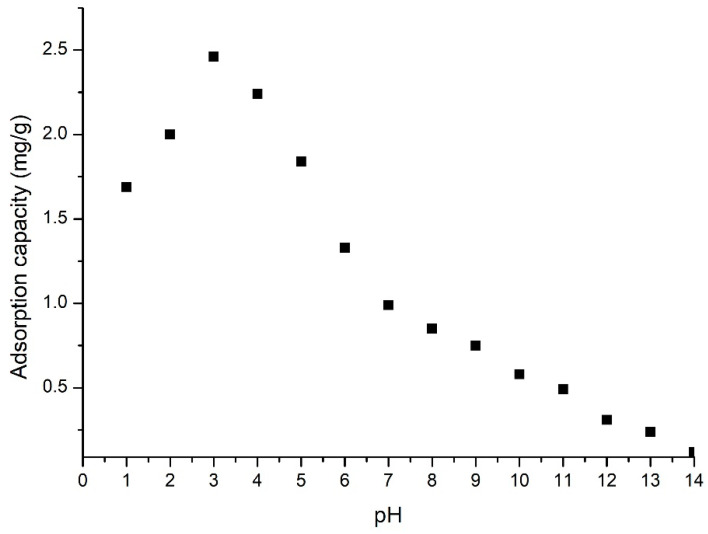
The pH influence.

Experimental data proved that the control parameter for the adsorption process is the pH, also this process is influenced by gold ionic form, and by the nature of functional groups existing on the adsorbent material surface. Taking these ideas into account, the optimum pH value for gold recovery from aqueous solutions by adsorption (obtained data being presented in Figure 8) was established.

The obtained experimental data prove that the pH increases until 3 leads to an increase in the material adsorption capacity, further increase of the pH leads to a decrease of the material adsorption capacity. A pH higher than 3 leads to a sharp decrease in the adsorption capacity, leading to no adsorption at higher pH. Based on this observation, we can conclude that the studied adsorption process can be conducted at a pH lower than 3 because the presence of HCl in the solution is favorable for the formation of chloro-anionic gold species [60,61].

#### 3.2.2. Contact Time and Temperature Effect

**Figure 9:**
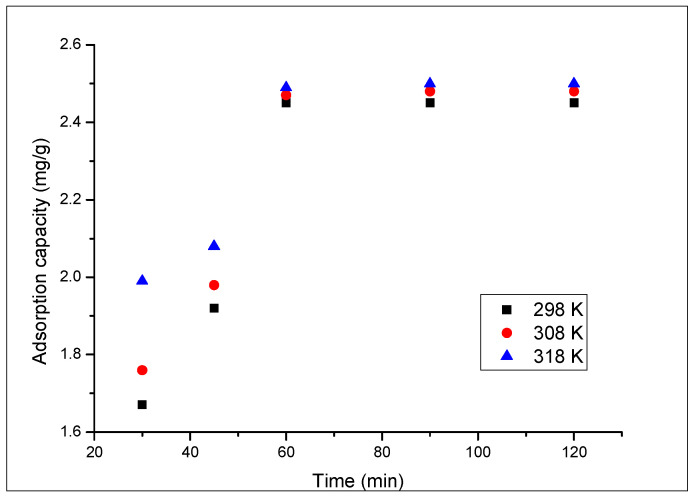
The contact time and temperature influence.

Figure 9 presents the contact time and temperature influence on the adsorption capacity of the prepared adsorbent material.

Based on the data depicted in Figure 9, we can observe that an increase of the contact time leads to an increase of the adsorption capacity of the adsorbent material. After 60 min, it is observed that the adsorption capacity reaches a value of 2.45 mg Au(III) per gram and then remains constant. Regarding the temperature influence, we can observe that the temperature increases from 298 to 318 K leads to a relatively low increase in the adsorption capacity (from 2.45 to 2.49 mg per gram of adsorbent material). Due to this low increase of the adsorption capacity, any further experiments were carried out at 298 K.

### 3.3. Kinetic, Thermodynamic, and Equilibrium Parameters for Adsorption Process

#### 3.3.1. Kinetic Study

**Figure 10:**
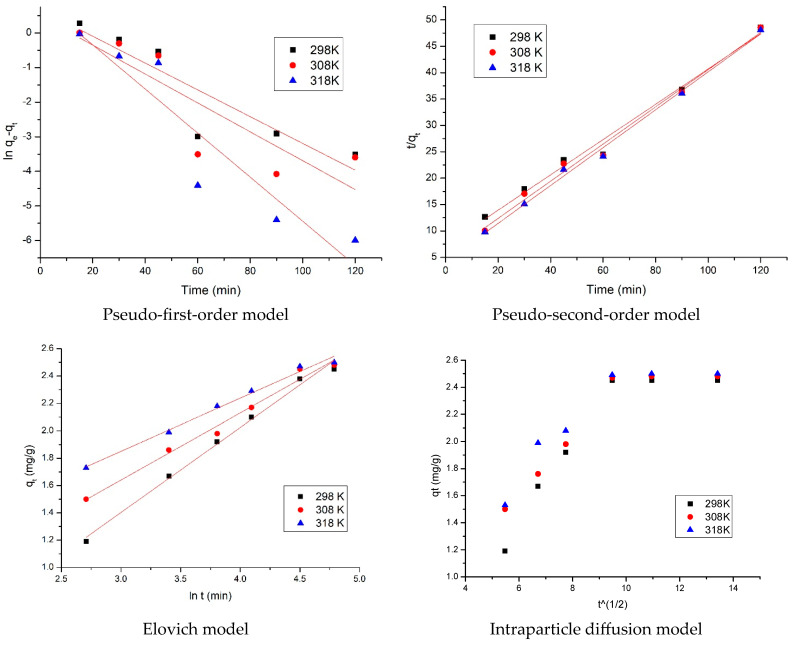
Kinetic, Elovich, and intraparticle diffusion models.

In order to analyze the kinetic gold adsorption process, and to really understand the kinetic mechanism which governs the process, obtained experimental data were modeled using three different kinetic models: pseudo-first-order kinetic model, pseudo-second-order kinetic model, and the intraparticulate diffusion model. Obtained isotherms are depicted in Figure 10. Based on these isotherms, the kinetic parameters for each model, were determined, and these parameters are presented in Table 3.

From the lines equation $\ln\left(q_{e}-q_{t}\right)=f(t)$ and $t/q_{t}=f(t)$ were calculated the speed constants k_1_ and k_2_ and the theoretical adsorption capacities associated with pseudo-first-order and pseudo-second-order kinetic models. Taking in account calculated kinetic parameters, we are able to establish which model is better at describing the gold adsorption on prepared adsorbent material. Regarding the gold adsorption on newly prepared adsorbent material, we can observe that the pseudo-second-order model is better at describing it. To distinguish between if film diffusion or intraparticulate diffusion represent the speed determining stage, obtained experimental data were modeled using the Weber and Morris model. In this way, it can be established if the adsorption process is taking place in several stages based on graphical representation of qt = f(t^1/2^). From this representation were calculated parameters $K_{diff}$ and C. 

From data depicted in Table 3, we can observe that the studied adsorption process is taking place in two stages. Also, the calculated $K_{diff}$ increases with the temperature increase. Diffusion constants associated with the first stage are much higher than those calculated for the second stage, meaning that the second stage is the speed determining stage [62]. When the experimental data were modeled using the Elovich model, it can be observed that the temperature increase leads to an increase of the α constant concomitant with a decrease of β constant. The α constant is related with the chemisorption rate, whereas the β constant is related with the surface coverage according to Teng and Hsieh [49,50]. From the obtained values of these parameters can concluded that the temperature increase leads to an increase of the chemical adsorption of gold on newly prepared adsorbent material.

#### 3.3.2. Thermodynamic Study

In order to obtain data associated with the energy changes associated with the studied process, thermodynamic studies into the temperature range 298 to 318 K were performed. Based on obtained thermodynamic data, it can be determined if the studied process is spontaneous or not, and if it is endo or exo-thermal. In this case, the values were calculated for $\Delta H^{0}$, $\Delta G^{0}$, and $\Delta S^{0}$.

**Figure 11:**
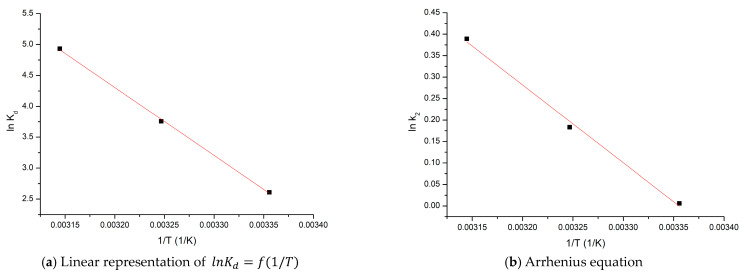
Thermodynamic studies.

Free entropy and free enthalpy values were calculated from graphical representation $lnK_{d}=f(1/T)$ (depicted in Figure 11a). Further, the values for free Gibbs energy variation were determined using the van’t Hoff equation.

Because chemical reactions play an important role in controlling the adsorption process speed, we must evaluate the minimum energy needed for transformation of reactants into the products. Based on graphical representation of Arrhenius dependence (data depicted in Figure 11b), we calculated the value of the activation energy needed for gold ions adsorption.

Table 4 presents calculated thermodynamic parameters associated with the studied adsorption.

The energy required to put the adsorbate in contact with the adsorbent is represented by the positive value of standard enthalpy ($\Delta H^{0}$). Because standard enthalpy variation has a positive value higher than 50 kJ/mol, we can conclude that, in this case, chemical bonds are formed between the adsorbate and the adsorbent surface [63].

Negative value of free Gibbs energy means that studied adsorption is a spontaneous and natural process. Adsorption process speed at the interface adsorbent/solution is correlated with the positive value of the standard entropy variation. 

#### 3.3.3. Activation Energy

Activation energy represent the minimum quantity of energy required to initiate a process. Based on Arrhenius equation, reaction speed is temperature dependent, meaning that by temperature increase, the process speed is also increasing [64].

For studied case, in first moment when the adsorbent and adsorbate are brought into contact, a limited number of collisions are taking place. This observation can be correlated with a low value of activation energy. In order to facilitate gold adsorption, it is required to bypass the free energy of the system. Because the studied process is endothermal one, it is recommended to introduce some energy into the system. This energy can increase the collision number between the adsorbate and adsorbent molecules, increasing simultaneously interaction forces [63]. Because the activation energy has a value of 15.1 kJ mol^−1^, can conclude that the studied adsorption process is a physical–chemical process [64]. 

#### 3.3.4. Equilibrium Study: Adsorption Isotherms

**Figure 12:**
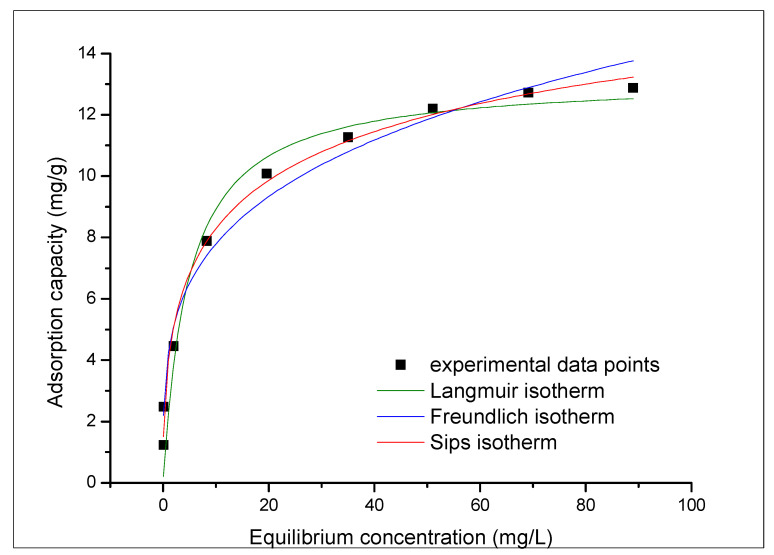
Adsorption isotherms.

In order to identify the adsorption mechanism, it is necessary to describe how the solution interacts with the adsorbent. This, can be obtained based on equilibrium isotherms which present the dependence between quantity of adsorbed ions per gram of adsorbent at equilibrium ($q_{e}$) and the residual concentration of gold ions into the aqueous phase ($C_{e}$). In this case, experimental data were modeled using Langmuir, Freundlich, and Sips isotherms (data depicted in Figure 12). Parameters associated with used isotherms are depicted in Table 5.

Based on data depicted in Table 5, we can conclude that gold recovery by an adsorption process is better described by the Sips isotherm due to the almost 1 value of the correlation coefficient, $R^{2}$. Also, by comparing the calculated adsorption capacity (13.1 mg g^−1^) with the experimental capacity (12.7 mg g^−1^), we can conclude that the Sips model is better to describe the studied adsorption process. From the value of the heterogeneity factor (n_s_ < 1), we can conclude that the gold adsorption on prepared material is a heterogeneous process [65].

Table 6 presents a comparison with other adsorbent materials used for gold recovery from aqueous solutions.

#### 3.3.5. Regeneration Degree of Adsorbent

**Figure 13:**
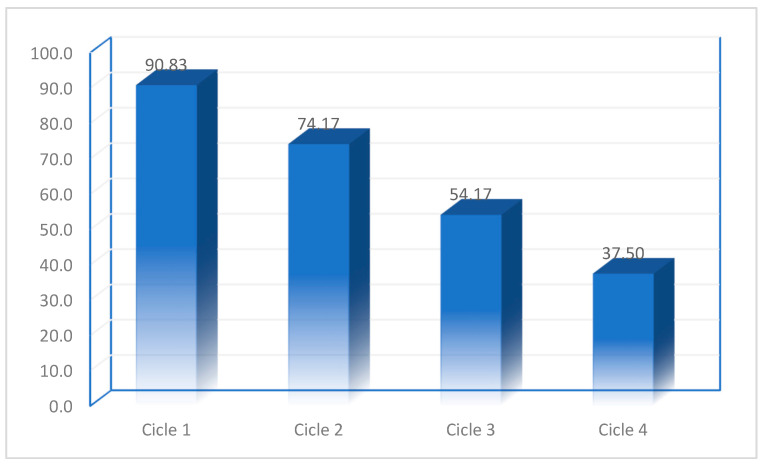
Regeneration degree of adsorbent.

In order to have an optimum price for the gold recovery process, it is important to produce and use an adsorbent material which can be reused as many times as possible. In this context, an important parameter is represented by the desorption capacity of the adsorbent material. Desorption studies proved if the prepared material has a practical process applicability in column adsorption studies [70,71]. Regarding gold adsorption on BCb/BC prepared material, all desorption studies were carried out using a 5% HCl solution. After the adsorption–desorption studies, we can conclude that the prepared material can be reused four times, as can be observed from data presented in Figure 13. The data presented in this figure shows that the desorption degree for prepared adsorbent materials varies between 90.8 and 37.5%.

#### 3.3.6. Material Usage in Case of Real Solution

Further, new prepared adsorbent material was used for gold recovery from real wastewater from an electronic industry. This study is important because real water contains Ni (II), Cu (II), and Fe (III) ions, so concomitant with gold recovery efficiency was evaluated with the material selectivity. In such wastewater, gold is present in the form of a K[Au(CN)2] complex, and it was important to brake it by using HCl and HNO_3_. Finally, a solution containing 60 mg Au(III)/L, 35 mg Ni(II)/L, 1 mg Cu(II)/L, and 2 mg Fe(III)/L was obtained. All experiments were carried out as previous described, obtaining a gold recovery efficiency of 90.5%. Together, after measuring the residual concentrations of other ions from filtered solution, it was observed that the process selectivity was 95%. Taking in account these observations, we can conclude that the newly prepared adsorbent material can be successfully used for gold recovery from real wastewaters.

## 4. Conclusions

Due to the simplicity and relatively low price, adsorption is preferred for water decontamination and for recovery of useful/secondary compounds from aqueous solutions. Adsorbent material selection depends on the nature and concentration of useful compound desired to be recovered, process global efficiency, and adsorption capacity of adsorbent material. 

A solid material with a higher adsorption capacity, but with low adsorption speed is not a good choice because it will require a longer contact time to allow for the penetration of the ions inside of the material. Similarly, a material with high adsorption speed, but lower adsorption capacity is not recommended due to the necessity to use higher quantities, which leads to a price increase.

An ideal adsorbent must have a higher adsorption capacity and present a higher adsorption speed. In this context, by using a sol-gel method, a new adsorbent material was prepared using bismuth carbonate (III) as a precursor. Black carbon powder was introduced into the adsorbent matrix. After preparation, all samples were characterized by specific physical–chemical techniques. Based on SEM and AFM analysis, it was observed that the material is formed from a particle agglomeration, with each particle having a dimension of around 200 nm. Further, from BET, it was observed that the produced material presents a specific surface of 41 m^2^ g^−1^ and pore dimensions around 11 nm. The recorded XRD spectrum showed the presence of a crystalline structure specific to bismuth oxide.

All adsorption studies presented in this paper were carried out in static conditions. The first part of the present study aimed to determine the influence of different parameters (pH, contact time, temperature, and initial concentration) on the adsorption process. The obtained experimental data showed that gold adsorption on BCp/BC material proceeds with a higher adsorption capacity when the solution pH is lower than 4. Regarding the contact time, it was observed that the optimum contact time is 60 min, and any further increase leads to no increase of the adsorption capacity. Temperatures have a positive effect on the studied adsorption, but the increase of the adsorption capacity does not justify the increase of the working temperature. The adsorption process obtained a maximum adsorption capacity of 12.7 mg Au(III) per gram of adsorbent material, for an initial concentration of Au(III) ions of 120 mg per litter.

Desorption studies proved the practical applicability of the prepared material which can be used for at least four adsorption–desorption cycles. Desorption degree of BCb/BC material varies from 90.8% in first cycle to 37.5% in last cycle.

Kinetic, thermodynamic, and equilibrium studies were performed to establish the mechanism of the studied adsorption process. The kinetic studies proved that the pseudo-second order kinetic model better describes gold adsorption on BCb/BC adsorbent material. This conclusion was obtained after taking into account the value of the correlation coefficient (value close to the unity) and the value of calculated adsorption capacity, which was close to the value obtained from the experiments.

The obtained experimental data was evaluated based on the value of the activation energy (15,1 kJ mol^−1^) for the gold adsorption process on BCb/BC prepared material. The experimental data are well modeled by Sips adsorption isotherm due to the regression coefficient close to unity. Thermodynamic studies proved that the gold recovery process by adsorption is a spontaneous and endothermal proess. It was also demonstrated that the studied adsorption is a physical–chemical process which takes place at the solid–liquid interface.

## Figures and Tables

**Table 1:** Textural parameters of sample BCb/CB.

Surface Area, m^2^/g	BJH Desorption, nm	Average Pore Diameter nm	Total Pore Volume, cc/g
41.00	3.95	11.48	1.126 × 10^−1^ cc/g for pores smaller than 137.1 nm

**Table 2:** Values obtained from AFM analysis.

Sample	Material Area (µm^2^)	Sa (µm)	Sq (µm)	Sp (µm)	Sv (µm)	Sy (µm)	Ssk	Sku
BCp/BC	114.388	0.179	0.237	0.677	−0.776	1.454	−0.273	3.529

**Table 3:** Kinetic parameters for the adsorption of Au (III) onto BCb/BC.

Pseudo-First Order				
Temperature (K)	$q_{e,exp}$ (mg g^−1^)	$k_{1}$ (min^−1^)	$q_{e,calc}$ (mg g^−1^)	$R^{2}$
298	2.45	0.0387	2.19	0.7911
308	2.48	0.0399	2.57	0.7542
318	2.50	0.0498	2.60	0.8753
Pseudo-second order				
Temperature (K)	$q_{e,exp}$ (mg g^−1^)	$k_{2}$ (g mg^−1^∙min^−1^)	$q_{e,calc}$ (mg g^−1^)	$R^{2}$
298	2.45	1.0060	2.49	0.9961
308	2.48	1.2010	2.48	0.9935
318	2.50	1.4760	2.51	0.9948
Elovich Model				
Temperature (K)	α (mg g^−1^ min^−1^)	β (mg g^−1^ min^−1^)	$R^{2}$	
298	1.61	3.38	0.9911	
308	2.04	1.44	0.9811	
318	2.57	0.51	0.9880	
Intraparticle diffusion model (IPD)				
Temperature (K)	$K_{diff}$ (mg·g^−1^ min^−1/2^)	C	$R^{2}$	
298	0.158	0.597	0.7311	
308	0.161	0.929	0.7675	
318	0.185	1.126	0.7131	

**Table 4:** Thermodynamic parameters for adsorption of Au (III) onto BCb/BC.

${\Delta H}^{0}$ (kJ/mol)	${\Delta S}^{0}$ (J/mol·K)	${\Delta G}^{0}$ (kJ/mol)			$R^{2}$
91.4	328.4	298 K	308 K	318 K	0.9993
		−6.42	−9.70	−12.99	

**Table 5:** Parameters of isotherm model for adsorption of of Au (III) onto BCb/BC.

Langmuir Isotherm			
$q_{m,exp}$ (mg/g)	$K_{L}$ (L/mg)	$q_{L}$ (mg/g)	$R^{2}$
12.70	0.290	13.20	0.9506
Freundlich Isotherm			
$K_{F}$ (mg/g)	1/nF		$R^{2}$
4.28	0.26		0.9769
Sips Isotherm			
$K_{s}$	$q_{s}$ (mg/g)	1/nS	$R^{2}$
0.56	13.10	0.21	0.9986

**Table 6:** Comparisons with other materials with adsorbent properties in order to recover Au(III) from aqueous solutions.

Material	Adsorption Capacity for Au(III) Recovery by Adsorption Process, mg/g	References
Nanometer TiO_2_ immobilized on silica gel	3.56	[66]
2-Mercaptobenzothiazole-bonded silica gel	4.50	[67]
Nanoparticles/graphitic carbon	7.92	[68]
Activated carbon	between 6 and 30, based on AC type	[69]
BCb/BC	12.70	This paper

## References

[1] Negrea A., Ronka S., Ciopec M., Duteanu N., Negrea P., Mihailescu M. (2021). Kinetics, Thermodynamics and Equilibrium Studies for Gold Recovery from Diluted Waste Solution. Materials.

[2] Reith F., Rea M.A.D., Sawley P., Zammit C.M., Nolze G., Reith T., Rantanen K., Bissett A. (2018). Biogeochemical cycling of gold: Transforming gold particles from arctic Finland. Chem. Geol..

[3] Philips G.N. Conglomerate-hosted gold deposits: What is so special? In Proceedings of the Gold18@Perth, An Australian Institute of Geoscientists symposium organised in conjunction with Geoscientists Symposia, Perth, WA, Australia, 2–3 August 2018.

[4] Saradesh K.M., Vinodkumar G.S. (2020). Metallurgical processes for hardening of 22Karat Gold for light weight and high strength jewelry manufacturing. J. Mater. Res. Technol..

[5] Corti C.W. (1999). Metallurgy of Microalloyed 24 Carat Golds. Gold Bull..

[6] Tamai T., Yamakawa M. (2017). Contact Resistance Property of Gold Plated Contact Covered with Contact Lubricant Under High Temperature. IEICE Trans. Electron..

[7] Côrtes L.N., Tanabe E.H., Bertuol D.A., Dotto G.L. (2015). Biosorption of gold from computer microprocessor leachate solutions using chitin. Waste Manag..

[8] Wang R., Xu Z. (2014). Recycling of non-metallic fractions from waste electrical and electronic equipment (WEEE): A review. Waste Manag..

[9] Wong M.H., Wu S.C., Deng W.J., Yu X.Z., Luo Q., Leung A.O.W., Wong C.S.C., Luksemburg W.J., Wong A.S. (2007). Export of toxic chemicals—A review of the case of uncontrolled electronic-waste recycling. Environ. Pollut..

[10] Syed S. (2006). A green technology for recovery of gold from non-metallic secondary sources. Hydrometallurgy.

[11] Ding Y., Zhang S., Liu B., Zheng H., Chang C.-C., Ekberg C. (2019). Recovery of precious metals from electronic waste and spent catalysts: A review. Resour. Conserv. Recycl..

[12] Hoffmann J.E. (1992). Recovering precious metals from electronic scrap. Jom.

[13] Lee J.-C., Song H.T., Yoo J.-M. (2007). Present status of the recycling of waste electrical and electronic equipment in Korea. Resour. Conserv. Recycl..

[14] Sum E.Y.L. (1991). The recovery of metals from electronic scrap. Jom.

[15] Ghosh B., Ghosh M.K., Parhi P., Mukherjee P.S., Mishra B.K. (2015). Waste Printed Circuit Boards recycling: An extensive assessment of current status. J. Clean. Prod..

[16] Morin D., Lips A., Pinches T., Huisman J., Frias C., Norberg A., Forssberg E. (2006). BioMinE—Integrated project for the development of biotechnology for metal-bearing materials in Europe. Hydrometallurgy.

[17] Andrews D., Raychaudhuri A., Frias C. (2000). Environmentally sound technologies for recycling secondary lead. J. Power Sources.

[18] Chmielewski A.G., Urbański T.S., Migdał W. (1997). Separation technologies for metals recovery from industrial wastes. Hydrometallurgy.

[19] Quinet P., Proost J., Van Lierde A. (2005). Recovery of precious metals from electronic scrap by hydrometallurgical processing routes. Min. Metall. Explor..

[20] Sheng P.P., Etsell T.H. (2007). Recovery of gold from computer circuit board scrap using aqua regia. Waste Manag. Res..

[21] Fleming C.A. (1992). Hydrometallurgy of precious metals recovery. Hydrometallurgy.

[22] Pilśniak M., Trochimczuk A.W., Apostoluk W. (2009). The Uptake of Gold(I) from Ammonia Leaching Solution by Imidazole Containing Polymeric Resins. Sep. Sci. Technol..

[23] Udayakumar S., Bin Abd Razak M.I., Ismail S. Recovering valuable metals from Waste Printed Circuit Boards (WPCB): A short review. Proceedings of the 14th AUN/SEED-Net Regional Conference on Materials (RCM) and 4th International Postgraduate Conference on Materials, Minerals and Polymer (MAMIP).

[24] Duong D.D. (1998). Adsorption Analysis: Equilibria and Kinetics. Series on Chemical Engineering.

[25] El-Naas M., Alhaija M.A. (2013). Modeling of Adsorption Processes, Mathematical Modelling.

[26] Lahuri A.H., Adnan R., Mansor M., Farah N., Nordin N. (2020). Adsorption Kinetics for Carbon dioxide Capture using Bismuth(III) Oxide Impregnated on Activated Carbon. Malays. J. Chem..

[27] Hakim A., Abu Tahari M.N., Marliza T.S., Wan Isahak W.N.R., Yusop M.R., Mohamed Hisham M.W., Yarmoa M.A. (2015). Study of CO2 adsorption and desorption on activated carbon supported iron oxide by temperature programmed desorption. J. Teknol..

[28] Yoshikawa K., Sato H., Kaneeda M., Kondo J.N. (2014). Synthesis and analysis of CO2 adsorbents based on cerium oxide. J. CO2 Util..

[29] Hakim A., Marliza T.S., Abu Tahari N.M., Isahak R., Yusop R.M., Hisham W.M.M., Yarmo A.M. (2016). Studies on CO2 Adsorption and Desorption Properties from Various Types of Iron Oxides (FeO, Fe2O3, and Fe3O4). Ind. Eng. Chem. Res..

[30] Rosynek M.P., Magnuson D.T. (1977). Infrared study of carbon dioxide adsorption on lanthanum sesquioxide and trihydroxide. J. Catal..

[31] Okawa Y., Tanaka K.-I. (1995). STM investigation of the reaction of Ag O added rows with CO2 on a Ag (110) surface. Surf. Sci..

[32] Takahashi H., Yuki K., Nitta T. (2002). Chemical modification of rutile TiO2(110) surface by ab initio calculations for the purpose of CO2 adsorption. Fluid Phase Equilibria.

[33] Isahak W.N.R.W., Ramli Z.A.C., Ismail M.W., Ismail K., Yusop R.M., Hisham M.W.M., Yarmo M.A. (2013). Adsorption–desorption of CO2 on different type of copper oxides surfaces: Physical and chemical attractions studies. J. CO2 Util..

[34] Hess G., Froitzheim H., Baumgartner C. (1995). The adsorption and catalytic decomposition of CO2 on Fe (111) surfaces studied with high resolution EELS. Surf. Sci..

[35] Bagherisereshki E., Tran J., Lei F., AuYeung N. (2018). Investigation into SrO/SrCO3 for high temperature thermochemical energy storage. Sol. Energy.

[36] Lahuri A.H., Abu Tahari M., Isahak W., Yusop R., Wahab M., Yarmo A. (2014). Temperature Programmed Desorption of Carbon Dioxide for Activated Carbon Supported Nickel Oxide: The Adsorption and Desorption Studies. Adv. Mater. Res..

[37] Ma W., Wang N., Lu Y., Lu Z., Tang X., Li S. (2019). Synthesis of magnetic biomass carbon-based Bi2O3 photocatalyst and mechanism insight by a facile microwave and deposition method. New J. Chem..

[38] Karnan T., Selvakumar S.A.S., Adinaveen T., Suresh J. (2016). Visible light induced photocatalytic degradation of azo dye by Bi_2_O_3_ nanoparticles synthesized using greener route. Int. J. Sci. Eng. Res..

[39] Ayekoe P.Y., Robert D., Gone D.L. (2018). Facile synthesis of TiO_2_/Bi_2_O_3_ heterojunctions for the photocatalytic degradation of water contaminants. J. Mater. Environ. Sci..

[40] Al-Ghouti M.A., Da’ana D.A. (2020). Guidelines for the use and interpretation of adsorption isotherm models: A review. J. Hazard. Mater..

[41] Yagub M.T., Sen T.K., Afroze S., Ang H.M. (2014). Dye and its removal from aqueous solution by adsorption: A review. Adv. Colloid Interface Sci..

[42] Namal O.O., Kalipci E. (2019). Adsorption kinetics of methylene blue using alkali and microwave-modified apricot stones. Sep. Sci. Technol..

[43] Lagergren S. (1898). About the theory of so-called adsorption of soluble substabces. Kungl. Sven. Vetensk. Handl..

[44] Ho Y.S. (2006). Review of second-order models for adsorption systems. J. Hazard. Mater..

[45] Ho Y.S., Mckay G. (1998). The kinetics of sorption of basic dyes from aqueous solution by sphagnum moss peat. Can. J. Chem. Eng..

[46] Weber W.J., Morris J.C. (1963). Kinetics of Adsorption on Carbon from Solution. J. Sanit. Eng. Div..

[47] Weber W.J., Morris J.C. (1964). Equilibria and Capacities for Adsorption on Carbon. J. Sanit. Eng. Div..

[48] Ebelegi A., Nimibofa A., Donbebe W. (2020). Interpretation of Adsorption Thermodynamics and Kinetics. Open J. Phys. Chem..

[49] Teng H., Hsieh C.-T. (1999). Activation Energy for Oxygen Chemisorption on Carbon at Low Temperatures. Ind. Eng. Chem. Res..

[50] Cheung C.W., Porter J.F., McKay G. (2001). Sorption kinetic analysis for the removal of cadmium ions from effluents using bone char. Water Res..

[51] Atkins P., de Paula J. (2005). Atkins’ Physical Chemistry.

[52] Fujiwara K., Ramesh A., Maki T., Hasegawa H., Ueda K. (2007). Adsorption of platinum (IV), palladium (II) and gold (III) from aqueous solutions onto l-lysine modified crosslinked chitosan resin. J. Hazard. Mater..

[53] Iftekhar S., Srivastava V., Sillanpää M. (2017). Synthesis and application of LDH intercalated cellulose nanocomposite for separation of rare earth elements (REEs). Chem. Eng. J..

[54] Zhang L., Zeng Y., Cheng Z. (2016). Removal of heavy metal ions using chitosan and modified chitosan: A review. J. Mol. Liq..

[55] Langmuir I. (1918). The adsorption of gases on plane surfaces of glass, mica and platinum. J. Am. Chem. Soc..

[56] Freundlich H.M.F. (1906). Over the adsorption in solution. J. Phys. Chem..

[57] McKay G., Otterburn M.S., Sweeney A.G. (1980). The removal of colour from effluent using various adsorbents—III. Silica: Rate processes. Water Res..

[58] Sips R. (1948). On the Structure of a Catalyst Surface. J. Chem. Phys..

[59] Ayawei N., Ebelegi A.N., Wankasi D. (2017). Modelling and Interpretation of Adsorption Isotherms. J. Chem..

[60] Mihăilescu M., Negrea A., Ciopec M., Davidescu C.M., Negrea P., Duţeanu N., Rusu G. (2019). Gold (III) adsorption from dilute waste solutions onto Amberlite XAD7 resin modified with L-glutamic acid. Sci. Rep..

[61] Chassary P., Vincent T., Sanchez Marcano J., Macaskie L.E., Guibal E. (2005). Palladium and platinum recovery from bicomponent mixtures using chitosan derivatives. Hydrometallurgy.

[62] Zhang S., Ning S., Liu H., Zhou J., Wang S., Zhang W., Wang X., Wei Y. (2020). Highly-efficient separation and recovery of ruthenium from electroplating wastewater by a mesoporous silica-polymer based adsorbent. Microporous Mesoporous Mater..

[63] Iftekhar S., Srivastava V., Sillanpää M. (2017). Enrichment of lanthanides in aqueous system by cellulose based silica nanocomposite. Chem. Eng. J..

[64] Brunauer S., Emmett P.H., Teller E. (1938). Adsorption of Gases in Multimolecular Layers. J. Am. Chem. Soc..

[65] Kumara N.T.R.N., Hamdan N., Petra M.I., Tennakoon K.U., Ekanayake P. (2014). Equilibrium Isotherm Studies of Adsorption of Pigments Extracted from Kuduk-kuduk (*Melastoma malabathricum* L.) Pulp onto TiO_2_ Nanoparticles. J. Chem..

[66] Liu R., Liang P. (2007). Determination of gold by nanometer titanium dioxide immobilized on silica gel packed microcolumn and flame atomic absorption spectrometry in geological and water samples. Anal. Chim. Acta.

[67] Pu Q.S., Su Z.X., Hu Z.H., Chang X.J., Yang M. (1998). 2-mercaptobenzothiazole-bonded silica gel as selective adsorbent for preconcentration of gold, platinum and palladium prior to their simultaneous inductively coupled plasma optical emission spectrometric determination. J. Anal. At. Spectrom..

[68] Wang L., Tian C., Mu G., Sun L., Zhang H., Fu H. (2012). Magnetic nanoparticles/graphitic carbon nanostructures composites: Excellent magnetic separable adsorbents for precious metals from aqueous solutions. Mater. Res. Bull..

[69] Soleimani M., Kaghazchi T. (2008). Adsorption of gold ions from industrial wastewater using activated carbon derived from hard shell of apricot stones—An agricultural waste. Bioresour. Technol..

[70] Vilvanathan S., Shanthakumar S. (2017). Column adsorption studies on nickel and cobalt removal from aqueous solution using native and biochar form of Tectona grandis. Environ. Prog. Sustain. Energy.

[71] Abdolali A., Ngo H.H., Guo W., Zhou J.L., Zhang J., Liang S., Chang S.W., Nguyen D.D., Liu Y. (2017). Application of a breakthrough biosorbent for removing heavy metals from synthetic and real wastewaters in a lab-scale continuous fixed-bed column. Bioresour. Technol..

